# Defining Priority Areas for Critical Care Simulation: A Modified Delphi Consensus Project

**DOI:** 10.7759/cureus.15844

**Published:** 2021-06-22

**Authors:** Ilana Harwayne-Gidansky, Adrian Zurca, Tensing Maa, Utpal S Bhalala, Deepa Malaiyandi, Pooja Nawathe, Aarti Sarwal, Muhammad Waseem, Michael Kenes, Megan Vennero, Lillian Emlet

**Affiliations:** 1 Pediatrics, Stony Brook Children’s Hospital, Stony Brook, USA; 2 Pediatric Critical Care Medicine, Penn State Health Children’s Hospital, Hershey, USA; 3 Pediatric Critical Care Medicine, Nationwide Children’s Hospital, Ohio State University College of Medicine, Columbus, USA; 4 Pediatric Critical Care Medicine, Children’s Hospital of San Antonio, San Antonio, USA; 5 Neuro-critical Care, University of Toledo, Toledo, USA; 6 Pediatric Critical Care Medicine, Cedars-Sinai Medical Center, Los Angeles, USA; 7 Neurology, Wake Forest School of Medicine, Winston-Salem, USA; 8 Pediatric Emergency Medicine, Lincoln Medical & Mental Health Center, New York, USA; 9 Pharmacy, University of Michigan, Ann Arbor, USA; 10 Emergency Medicine, Veterans Affairs Pittsburgh Healthcare System, Pittsburgh, USA; 11 Emergency Medicine, University of Pittsburgh Medical Center, Pittsburgh, USA

**Keywords:** interprofessional education and collaboration, simulation, medical education, training, pulmonary critical care, critical care, pediatric emergencies and critical care

## Abstract

Background

Simulation is used in critical care for skill development, formative assessment, and interprofessional team performance. Healthcare educators need to balance the relatively high cost to deliver simulation education with the potential impact on healthcare quality. It is unclear how to prioritize simulation in critical care education, especially considering interprofessional needs across adult and pediatric populations. The objective of this study was to prioritize topics for critical care educators developing simulation-based educational interventions.

Methodology

A modified Delphi process was used to identify and prioritize critical care topics taught using simulation. We disseminated a multi-institutional survey to understand critical care simulation topics using a three-round modified Delphi technique. An expert panel was recruited based on their expertise with simulation-based education through the Society for Simulation in Healthcare and the Society of Critical Care Medicine lists. Critical care topics originated using content derived from multiple critical care board examination contents. Additional content for a critical care simulation-based curriculum was generated.

Results

Consensus and prioritization were achieved in three rounds, with 52 simulation experts participating. The first Delphi round surveyed priority topics in critical care content and generated additional topics for inclusion in round two. The second Delphi round added the content with the highest-ranked items from round one to generate a set of simulation-based topic priorities. The third Delphi round asked participants to determine the importance of each priority item taught via simulation compared to other modalities for clinical education. This round yielded 106 topics over four domains categorized into (1) Diagnosis and Management of Clinical Problems, (2) Procedural Skills, (3) Teamwork and Communication Skills, and (4) General Knowledge and Knowledge of Technical Adjuncts.

Conclusions

The modified Delphi survey revealed a prioritized, consensus-based list of topics and domains for critical care educators to focus on when creating a simulation-based critical care curriculum. Future work will focus on developing specific simulation-based critical care curricula.

## Introduction

Simulation-based medical education (SBME) is widely accepted as an effective method to teach interprofessional and interdisciplinary team communication skills, technical skills, high-stakes rare events, and integrative case-based demonstration of higher-level skills [1]. Healthcare professionals across the continuum need mastery of these skills and core competencies from undergraduate medical education (UME) to postgraduate training in multiple healthcare disciplines and specialties. In critical care, simulation has historically focused primarily on resuscitation and procedural skills.

Evidence supports using SBME to teach numerous domains of critical care, spanning across multiple medical specialties and healthcare professions [2-4]. Educators and healthcare systems must balance the financial and personnel costs of SBME with the effectiveness of reaching desired learning and healthcare outcomes [5]. Although SBME is resource-intensive, its integration in curricula has dramatically expanded its reach, demonstrating improvements to patient-driven outcomes [6-8]. These include just-in-time infant lumbar puncture, simulation-based mastery learning of central venous line (CVL) placement, and using virtual reality to teach patient assessments [9-13].

Despite the evidence of its importance, there is no current widespread standardized curriculum or guidance on critical care simulation content for educators. Although several disciplines have developed guidelines for teaching critical care topics, none have exclusively prioritized the role of a simulation-based curriculum for this level of intensive care training [14,15]. Therefore, it remains unclear how to prioritize topics best suited to be taught via simulation for critical care healthcare professionals. Thus, we aimed to assess the current status of SMBE in critical care and develop priority areas for the effective and efficient utilization of simulation for critical care education.

## Materials and methods

Design and Delphi process

**Figure 1:**
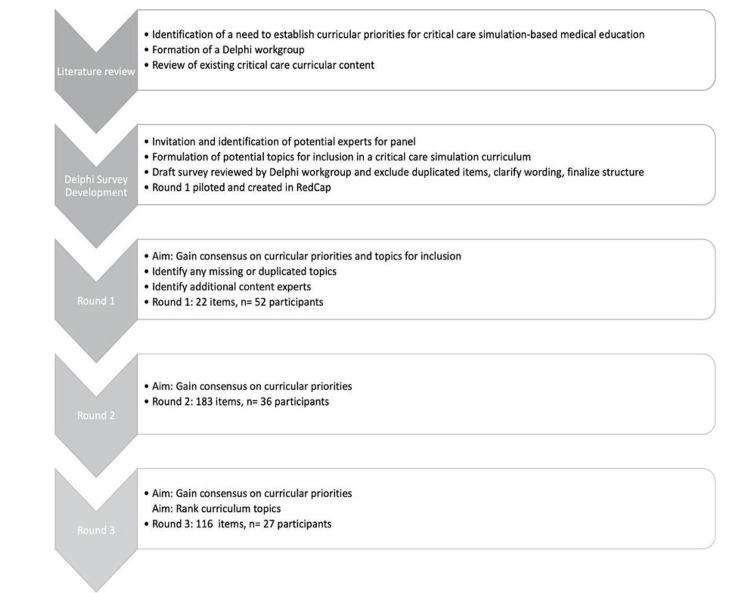
Methodological process for generating prioritized simulation-based critical care content.

A modified Delphi was used to develop consensus around SBME priorities and present a list of content to guide future simulation-based educational priorities such as generating a standard critical care simulation curriculum [14-16]. The Delphi method relies on expert opinion to arrive at these conclusions. A six-step approach is adopted when utilizing this process: (1) stating the problem, (2) developing an initial questionnaire, (3) selecting experts, (4) iteratively polling experts, (5) identifying potential consensus points, and (6) reporting results. This approach allows for an iterative understanding of the question with feedback given by experts between cycles [16-18] (Figure 1).

Identification of simulation education experts for Delphi panel

The research team was composed of interprofessional members of the Society of Critical Care Medicine (SCCM) Research Section, Simulation Education Research Subcommittee. Simulation education experts were identified through the purposeful sampling of publicly available lists of simulation centers to identify experts in critical care simulation education for this panel. Representatives of accredited simulation centers available through the American Board of Anesthesiology and Society for Simulation in Healthcare were approached, along with members of the SCCM Education Research Committee. We defined “critical care simulation educator” as someone who uses any modality of technology-enhanced simulation to educate learners, utilizing evidence-based strategies directed at critical care practice objectives [19]. We established a panel of 52 experts from multiple professions consistent with the standard Delphi procedure. A panel of interprofessional content experts included physicians, nurses, educators, and pharmacists. The snowball method was used to sample participants, where each participant was asked in the initial survey to identify additional expert participants who then also received an invitation to participate in the study [20]. Experts were sent an email invitation explaining the study’s purpose and a link to the survey for the first round of the Delphi. Participation was voluntary, and this study was approved by the University of Pittsburgh Institutional Review Board as a non-human subjects exempt survey research study. The participants remained anonymous to each other throughout the study.

Study data were collected and managed using REDCap electronic data capture tools co-hosted at the University of Pittsburgh and Stony Brook University. Surveys were distributed between December 2018 and June 2019. Responses were collected and de-identified for final data analysis. No limits on respondents were set a priori. Once panelists agreed to participate, a link to the first round of the survey was sent, along with detailed instructions on how to complete the Delphi surveys.

The writing group consisted of 12 members, a diverse interprofessional and interdisciplinary group of simulation educators and facilitators from neurocritical care (2), pediatric critical care (5), emergency medicine-critical care (1), pediatric emergency medicine (1), critical care nurse practitioners (1), and critical care pharmacist (1).

Survey design and iterative feedback

Initial first-round items were derived from blueprint content of critical care board examinations from the following specialties: the American Board of Internal Medicine, American Board of Surgery, American Board of Pediatrics, American Board of Emergency Medicine, and American Board of Anesthesiology. Descriptions of training program requirements in critical care medicine were reviewed to determine a list of topics for ranking in the survey. As all study team members were critical care simulation educators, a pilot test of the survey for readability, feasibility, and face validity was performed, and changes were made as needed. Experts were asked to prioritize each topic on a three-point Likert scale (least important, somewhat important, most important). Free text options to describe the current simulation curriculum and its associated objectives were included. At the end of the first round, unique free-text items were analyzed and included in the second round as potential additions to curricular content.

Consensus was defined a priori as >80% agreement with “somewhat” or “most” important selected. Topics with a consensus of <80% were not included in the priority list for round two as they were thought to be unlikely to reach a consensus. Round two items were aggregated from round one, and potential topics for inclusion in a critical care simulation content outline were included in the final round of content generation. At the end of the second round, we selected the items from the list of consensus curriculum that achieved this threshold [16-18,21]. Following this round, we formulated potential consensus items for inclusion into the third round. This method was used, in part, to assure a more comprehensive capture of content.

For round three, items were again aggregated and then prioritized into three domains of content that were most important, somewhat important, or least important to include as SBME content. For each ranking, we asked participants to rate each topic using a three-point Likert scale (with options labeled 1 = best taught via simulation to 3 = should not be taught via simulation).

To obtain a prioritized list of critical care content, we used the methods proposed by Altschuld and Thomas, where content is ranked and scored according to the frequency of occurrence for prioritization scoring [22]. According to this method, minimum priority was defined as a prioritization score of >50, with strong prioritization >60. Those topics not included in the priority list scored <50 were classified as low priority and not included.

Outcome measures

The primary outcomes of interest were ranked in order of importance of areas of critical care content. We defined “simulation education” as any modality of technology-enhanced (computer, manikin, standardized patient, excluding animal, tissue, or cadaveric models) simulation, which is an educational tool with which the learner interacts to mimic an aspect of clinical care for the purpose of teaching or assessment in the delivery of critical care to adult or pediatric patients [19]. The primary outcome of the third round Delphi was to generate a list of prioritized content deemed important by this multi-professional group of critical care simulation experts.

**Figure 2:**
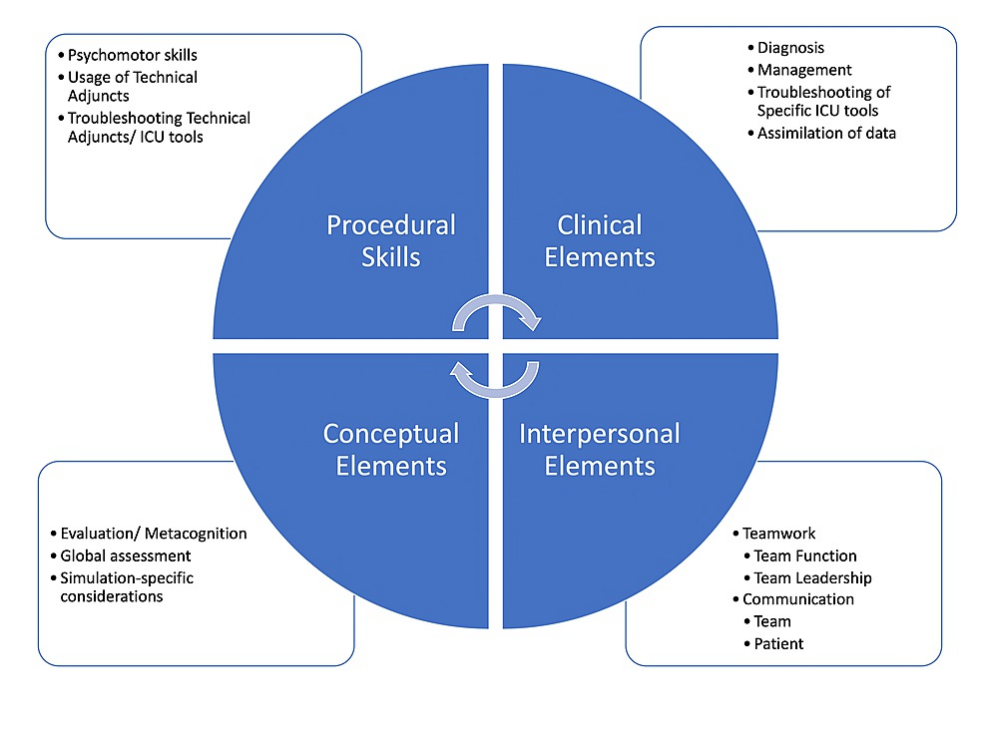
Priority domains for critical care simulation. ICU: intensive care unit

Consensus was reached after round three, from which we developed a critical care simulation content outline. All recommended topics were mapped into four main learning domains: General Knowledge, Diagnosis and Management, Procedural Skills, and Teamwork and Communication; consensus was reported among all simulation specialists (Figure 2).

## Results

A total of 52 experts participated in the initial survey, 27 of whom completed all three rounds (52%). The majority of participants worked in critical care. There were 34 (65%) respondents who were physicians, followed by nurse practitioners (n = 6, 12%), nurses (n = 5, 10%), pharmacists (n = 4, 8%), respiratory therapists (n = 2, 4%), and other (n = 1, 2%). Experts most commonly represented pediatrics (n = 14, 27%) and emergency medicine (n = 9, 17%). These experts reported working in 16 states across all geographical regions of the United States. Additional demographic data of the simulation experts are presented in Table 1.

**Table 1:** Demographic information.

		N = 52	(%)
Please list your primary patient population	Pediatric patients (including neonatology)	20	(41.6%)
	Adult patients	25	(52.0%)
	Other (please specify)	3	(6.3%)
Profession	Physician	34	(65.4%)
	Nurse	5	(9.6%)
	Respiratory therapist	2	(3.8%)
	Pharmacist	4	(7.7%)
	Nurse practitioner	6	(11.5%)
	Other (please specify)	1	(1.9%)
In which clinical area do you primarily work?	Neuro-intensive care	3	(5.8%)
	Emergency medicine	9	(17.3%)
	Cardiovascular intensive care	2	(3.9%)
	Trauma/surgical intensive care	5	(9.6%)
	Anesthesiology	3	(5.8%)
	Pediatrics	14	(26.9%)
	Other	7	(13.5%)
	Medical intensive care	5	(9.6%)
	Nonclinical	4	(7.7%)
How many years have you worked in your current profession (excluding trainee time)?	0-5	4	(7.7%)
	6-10	20	(38.5%)
	11-15	6	(11.5%)
	16-20	6	(11.5%)
	>20	15	(28.8%)

The first round of the Delphi survey contained demographic information and 22 items focused on simulation, including free-text boxes for participants to add additional critical care curriculum content not previously captured. The second survey included 183 questions and with a completion rate of 69% (36/52). The third and final survey generated a list of 116 elements of ranked content and was returned with a completion rate of 75% (27/36).

After the Delphi process, experts identified 106 items to be recommended for critical care content over four domains. Topics with a score of >60 were strongly prioritized in the four domains of General Knowledge and Knowledge of Technical Adjuncts, Diagnosis and Management, Procedural Skills, and Teamwork and Communication Skills, and are listed in Tables 2-5.

**Table 2:** Strongly prioritized topics: General Knowledge and Knowledge of Technical Adjuncts Domain.

Topic	Category theme	Score	Overall rank order
Extracorporeal life support	Procedural Skills	67	13
Intra-aortic balloon pump	Procedural Skills	67	13
Ventricular assist devices	Procedural Skills	67	13
Use of invasive neurologic monitoring (e.g., external ventricular drain and bolts)	Procedural Skills	64	16
Continuous renal replacement therapy usage and troubleshooting	Procedural Skills	62	18
Limitations of the simulation (e.g., proper pre-simulation preparation)	Conceptual Element	62	18
Surgical technical adjuncts	Procedural Skills	62	18
Understanding how to use and troubleshoot respiratory technological adjuncts (e.g., mechanical ventilation)	Procedural Skills	62	18
Utilization of hemodynamic monitoring data in patient management	Clinical Elements	62	18
Checking medications	Clinical Elements	61	19

**Table 3:** Strongly prioritized topics: Diagnosis and Management Domain.

Topic	Category theme	Score	Overall rank order
Cardiovascular/hemodynamic emergencies: management of patients in cardiac arrest	Clinical Elements	75	5
Diagnosis and management (e.g., clinical reasoning) during a code	Clinical Elements	73	7
Management of acute trauma resuscitation (e.g., blunt, penetrating)	Clinical Elements	73	7
Management of anaphylaxis	Clinical Elements	73	7
Management of bleeding emergencies (e.g., hemorrhage)	Clinical Elements	70	10
Diagnosis and management of shock states	Clinical Elements	68	12
Management of neurologic emergencies (e.g., intracerebral hemorrhage, status epilepticus, head trauma, spinal cord trauma)	Clinical Elements	68	12
Diagnosis of hyperthermic emergencies (i.e., malignant hyperthermia/neuroleptic malignant syndrome)	Clinical Elements	67	13
Cardiovascular diagnosis and management (e.g., arrhythmia, acute coronary syndrome, heart failure, pulmonary hypertension)	Clinical Elements	65	15
Cardiovascular/hemodynamic emergencies: management of patients in low cardiac output state (not cardiac arrest)	Clinical Elements	65	15
Management of infectious disease emergencies (e.g., septic shock)	Clinical Elements	65	15
Medical management of pericardial tamponade	Clinical Elements	65	15
Pulmonary emergencies (e.g., hemorrhage, edema)	Clinical Elements	63	17
Diagnosis and management of acute respiratory failure	Clinical Elements	62	18
Approach and management of bioterrorism	Clinical Elements	60	20
Gastrointestinal emergencies (e.g., gastrointestinal hemorrhage, esophageal perforation)	Clinical Elements	60	20

**Table 4:** Strongly prioritized topics: Procedures Domain.

Topic	Category theme	Score	Overall rank order
Physical performance during a code (such as chest compression or bag-valve mask quality)	Procedural Skills	79	1
Endotracheal intubation procedure	Procedural Skills	75	5
Placement of hemodynamic monitoring devices (central venous and arterial lines)	Procedural Skills	74	6
Mastery of a specific procedural skill	Procedural Skills	73	7
Procedural training: individual steps	Procedural Skills	73	7
Procedures: pericardiocentesis	Procedural Skills	73	7
Ability to repeat skill/procedure more than once	Procedural Skills	72	8
Establishing and maintaining a sterile field	Procedural Skills	71	9
Familiarity with equipment	Procedural Skills	70	10
Pleural drainage/chest tube placement	Procedural Skills	70	10
Abdominal paracentesis	Procedural Skills	69	11
General airway management procedures except endotracheal intubation	Procedural Skills	69	11
Regional anesthesia procedures (nerve blocks or epidural placement)	Procedural Skills	69	11
Swan Ganz placement	Procedural Skills	67	13
Understanding the importance of each step of a procedure	Conceptual Elements	67	13
Checklist usage for a procedure	Conceptual Elements	66	14
Neurologic (e.g., burr hole)	Procedural Skills	66	14
Use of point-of-care ultrasound	Procedural Skills	66	14
Troubleshooting during a procedure	Procedural Skills	65	15
Procedures: performing a timeout	Conceptual Elements	62	18

**Table 5:** Strongly prioritized topics: Teamwork and Communication Domain.

Topic	Category theme	Score	Overall rank order
Situational awareness during a code	Conceptual Elements	79	1
Speaking up during resuscitation	Interpersonal Elements	77	3
Team-specific general situational awareness	Interpersonal Elements	77	3
Error recovery during team training	Interpersonal Elements	76	4
Interprofessional team dynamics	Interpersonal Elements	76	4
Leadership development during a code	Interpersonal Elements	76	4
Team leader cognitive load during a code	Interpersonal Elements	76	4
Time management during mock codes	Interpersonal Elements	76	4
Algorithm application during a code (pediatric advanced life support, advanced cardiovascular life support)	Clinical Elements	75	5
Communicating bad news	Interpersonal Elements	75	5
Rapid decision-making during emergencies	Interpersonal Elements	75	5
Team training (e.g., team communication and coordination)	Interpersonal Elements	75	5
Interprofessional leadership	Interpersonal Elements	74	6
Understanding team roles and responsibilities including role identification and execution	Interpersonal Elements	74	6
Using standard communication tools (e.g., TeamSTEPPS, situation-background-assessment-recommendation/request)	Interpersonal Elements	74	6
Collaboration and sharing of concept knowledge during a code	Interpersonal Elements	73	7
Using debriefing as a teaching tool	Conceptual Elements	73	7
Error detection during a code	Conceptual Elements	72	8
Uncovering systems vulnerabilities during a code	Conceptual Elements	72	8
Ability to maintain a “big picture” view	Conceptual Elements	71	9
Avoidance of hierarchy issues	Interpersonal Elements	71	9
Avoiding cognitive bias when making diagnostic errors during a code	Conceptual Elements	71	9
Contingency planning during a code	Conceptual Elements	71	9
Debriefing after a code	Interpersonal Elements	71	9
Effective pre-briefing for codes	Interpersonal Elements	71	9
Patient assessment during a code	Clinical Elements	71	9
Avoidance of fixation	Conceptual Elements	70	10
Communicating empathy to patients and families	Interpersonal Elements	70	10
Confidence building for trainees during a code	Conceptual Elements	69	11
Competency managing and delegating procedures	Interpersonal Elements	68	12
Preparation during a code	Interpersonal Elements	68	12
Critical self-analysis during team training	Conceptual Elements	67	13
Effective knowledge and utilization of resources during team training	Interpersonal Elements	67	13
Site-specific planning during team training	Interpersonal Elements	66	14
Establishing patient-centered goals of care	Interpersonal Elements	62	18
Culture of empowerment	Interpersonal Elements	61	19
Team training for palliative care/end-of-life coordination/medical futility	Interpersonal Elements	61	19

Topics with moderate priority are listed in Table 6.

**Table 6:** Moderately prioritized topics.

Topic	Score	Overall rank order
Deep knowledge of critical illness (beyond algorithms)	57	23
Overdoses and poisonings: medication indications and side effects (of drug(s) taken)	57	23
Anesthesia medication indications and effects (e.g., analgesic, sedatives, neuromuscular blockade)	52	27
Cardiopulmonary interactions	52	27
General analgesia, sedation, neuromuscular blockade	51	28
Declaration and management of brain death	57	23
Diagnosis and management of burns and drownings	57	23
Managing environmental emergencies (e.g., drowning, burns)	57	23
Approach to palliative liberation of an endotracheal tube	55	24
Neurologic diagnosis and management (e.g., cerebrovascular disease, seizures)	55	24
Approach to ethical issues (e.g., conflict of interest, patient privacy)	54	25
Diagnosis and management of altered mentation or coma (e.g., encephalopathy, coma, delirium)	54	25
Managing endocrine emergencies (e.g., diabetic ketoacidosis, thyroid storm, hypocalcemia)	54	25
Toxic ingestions (diagnosis and management)	54	25
Diagnosis and management of bites and envenomations	53	26
Managing compartment syndrome/pulseless extremity	53	26
Management of genetic/metabolic emergencies (e.g., metabolic crisis)	52	27
Surgical diagnoses and management(e.g., abdominal compartment syndrome)	52	27
Obtaining consent for a procedure	59	21
Echocardiography	58	22

Evaluation of the recommended topics yielded four major domains: Procedural Skills, Clinical Elements, Conceptual Elements, and Interpersonal Elements (Figure 2).

## Discussion

We developed simulation-based critical care education priorities through the consensus of simulation experts across all pediatric and adult critical care facets. In defining these domains and categories of core simulation-based critical care content, we hope to improve the prioritization of SBME within critical care. Identifying priority areas for simulation-based education may help stakeholders, including healthcare organizations, professional societies, and educational leadership, prioritize funding, budget allocations, faculty/staff support, research collaboratives, and program assessments. This content should aid training programs and simulation centers in evaluating learners and target learning needs specific to critical care medicine.

Prior work using the Delphi process for content generation has been used to generate UME content for critical care [15] and pediatric content for emergency medicine residents [14]. In both cases, the scope was different and not adequately specific to apply to critical care core competencies in the context of either graduate medical education or interprofessional education, again highlighting a need for this study. Not surprisingly, our results demonstrate strong agreement that simulation is well suited to teach several aspects of communication and procedural skills. A focus on aspects of cardiac arrest, shock, and other resuscitation scenarios was also noted to be important components of SBME in intensive care units. This study also demonstrates a potential role for SBME to teach more cognitive skills such as diagnosis and management of various clinical entities.

A key finding of our study was a generally good agreement on the topics for critical care education across teamwork, communication, and procedural skills. While pathophysiology and diagnosis may differ across the age spectrum and between different medical and surgical subspecialties, often the basics of procedures and especially the skills necessary for effective communication during resuscitation events transcend specialties and professions. This suggests that combining interprofessional critical care simulation programs across subspecialties may benefit from optimizing resource utilization. We hope that this will ultimately facilitate a shift from training in silos to training together through interprofessional simulations. In doing so, we might better approximate how we might create better learning activities for critical care teams.

Despite an underrepresentation of neurointensivist participation, neurological emergencies were identified as an area of high priority, potentially reflecting emerging awareness of the utility of SBME for teaching about neurological emergencies and technological advances that support creating such simulations. Intensivists, including pediatric, emergency medicine, and trauma critical care providers, have essential roles in the initial identification and management of acute neurological injury and thus may have ranked these topics higher.

One of the main challenges of the widespread adoption of simulation education is insufficient faculty time and the limited ability to regularly pull trainees and interprofessional colleagues from clinical care responsibilities. Especially during the global coronavirus disease 2019 (COVID-19) pandemic, any disruption to the available pool of faculty and trainees available to provide bedside care is unlikely to be supported and may result in the cancellation of simulation courses altogether. By co-training learners from different specialties, no individual department is left with inadequate patient care coverage. Additionally, if course goals and objectives are uniform, faculty across different specialties could share the responsibility of teaching sessions. Finally, expanding the pool of potential learners and research subjects can further facilitate the performance of simulation education research. Additionally, the COVID-19 pandemic has shown the importance of cross-training healthcare providers who are not typically critical care providers for such surges. This priority list helps healthcare systems and simulation centers determine key objectives for rapid just-in-time training.

Our work has several significant limitations, including a variable survey response rate and drop-off between rounds resulting in a small sample size of predominantly physicians and a higher ratio of pediatric-to-adult intensivists. Additionally, it is not possible to extrapolate if the opinions expressed by educators truly reflect learners’ needs. Third, our results may have been different had this study been performed after the onset of the COVID-19 pandemic.

The Delphi method has inherent limitations within the process itself. The intention is to gather and obtain consensus based upon experts’ opinions and insight. It does not result in an evidence-based summary (a Delphi is performed precisely because of a lack of evidence within a specific area). Additionally, the final product is a list of statements that gained consensus but does not provide additional detail or depth to those answers. This study’s specific aims focused on overall expert opinion for which consensus was sought over a wide range of content, spanning multiple disciplines, several specialties, and age ranges, thus limiting the ability to obtain in-depth input for any specific subspecialty or pathology. However, the broad net that was cast in this project may also be seen as a strength as it increases the generalizability of the findings. Given the scope of the study, some of the topics listed by subspecialty boards were more detailed than others, leading to a wide range in the specificity of the topics included.

The Delphi survey’s extensive and iterative nature could have led to potential survey fatigue and content bias. The composition of our expert panel may have been biased as well. Although we tried to mitigate this by broadly recruiting experts in the field through multiple lists, the final panel was somewhat self-selecting by answering our recruitment emails. Given that this Delphi process was conducted remotely, as opposed to in-person, bias from a smaller group of “influential” experts was less likely to have occurred. However, using a remote method without an in-person component may have also contributed to the relatively high attrition rate experienced. While we had a reasonably sized group, it is unclear from existing literature what the ideal size of an expert panel should be. A group should generally be large enough to propose new ideas and overcome attrition issues. A total of 30 members have been suggested as a reasonable target to attain these goals and align with our final survey round [23]. Finally, while we sought to have a broad representation of experts across disciplines within the United States, these results lack international representation and likely differ in other countries, where variations in educational curricula, competing interests, and different priorities may be present.

## Conclusions

In conclusion, we defined priority areas in SBME curricula for critical care using a modified Delphi process. Healthcare systems and simulation centers should focus on these criteria when designing training curricula for critical care teams across medical professions and subspecialties and consider sharing resources between different groups of faculty and learners. As critical care practice changes with new medications, technology, and multispecialty teams, judicious use of simulation will help ensure timely and effective educational interventions that will also inform quality improvement and safety initiatives.

## References

[1] Lopreiato JO, Sawyer T (2015). Simulation-based medical education in pediatrics. Acad Pediatr.

[2] Hippe DS, Umoren RA, McGee A, Bucher SL, Bresnahan BW (2020). A targeted systematic review of cost analyses for implementation of simulation-based education in healthcare. SAGE Open Med.

[3] Kester-Greene N, Hall AK, Walsh CM (2019). [Simulation curricular content in postgraduate emergency medicine: a multicentre Delphi study]. CJEM.

[4] Bessmann EL, Østergaard HT, Nielsen BU (2019). Consensus on technical procedures for simulation-based training in anaesthesiology: a Delphi-based general needs assessment. Acta Anaesthesiol Scand.

[5] Doughty CB, Kessler DO, Zuckerbraun NS (2015). Simulation in pediatric emergency medicine fellowships. Pediatrics.

[6] Manthous CA (2014). On the outcome project. Yale J Biol Med.

[7] Seam N, Lee AJ, Vennero M, Emlet L (2019). Simulation training in the ICU. Chest.

[8] Swing SR (2007). The ACGME outcome project: retrospective and prospective. Med Teach.

[9] Kessler D, Pusic M, Chang TP (2015). Impact of just-in-time and just-in-place simulation on intern success with infant lumbar puncture. Pediatrics.

[10] Barsuk JH, McGaghie WC, Cohen ER, O'Leary KJ, Wayne DB (2009). Simulation-based mastery learning reduces complications during central venous catheter insertion in a medical intensive care unit. Crit Care Med.

[11] Barsuk JH, McGaghie WC, Cohen ER, Balachandran JS, Wayne DB (2009). Use of simulation-based mastery learning to improve the quality of central venous catheter placement in a medical intensive care unit. J Hosp Med.

[12] Cohen ER, Feinglass J, Barsuk JH, Barnard C, O'Donnell A, McGaghie WC, Wayne DB (2010). Cost savings from reduced catheter-related bloodstream infection after simulation-based education for residents in a medical intensive care unit. Simul Healthc.

[13] Zackoff MW, Real FJ, Sahay RD (2020). Impact of an immersive virtual reality curriculum on medical students' clinical assessment of infants with respiratory distress. Pediatr Crit Care Med.

[14] Mitzman J, King AM, Fastle RK (2017). A modified Delphi study for development of a pediatric curriculum for emergency medicine residents. AEM Educ Train.

[15] Smith AG, Brainard JC, Campbell KA (2020). Development of an undergraduate medical education critical care content outline utilizing the Delphi method. Crit Care Med.

[16] Humphrey-Murto S, Wood TJ, Gonsalves C, Mascioli K, Varpio L (2020). The Delphi method. Acad Med.

[17] Johnston L, Sawyer T, Nishisaki A (2019). Neonatal intubation competency assessment tool: development and validation. Acad Pediatr.

[18] Brown BB (1968). Delphi process: a methodology used for the elicitation of opinions of experts. https://www.rand.org/pubs/papers/P3925.html.

[19] Lioce L, Lopreiato J, Downing D (2020). Healthcare simulation dictionary. https://www.ahrq.gov/sites/default/files/wysiwyg/patient-safety/resources/simulation/sim-dictionary-2nd.pdf.

[20] Johnson TP (2014). Snowball sampling: introduction.

[21] Lynn MR (1986). Determination and quantification of content validity. Nurs Res.

[22] Altschuld JW, Thomas PM (2016). Considerations in the application of a modified scree test for Delphi survey data. Eval Rev.

[23] Murphy MK, Black NA, Lamping DL, McKee CM, Sanderson CF, Askham J, Marteau T (1998). Consensus development methods, and their use in clinical guideline development. Health Technol Assess.

